# Treatment of disseminated keratosis pilaris-associated itch with dupilumab

**DOI:** 10.1016/j.jdcr.2024.12.010

**Published:** 2024-12-23

**Authors:** Kelsey L. Auyeung, Brian S. Kim

**Affiliations:** a Kimberly and Eric J. Waldman Department of Dermatology, Icahn School of Medicine at Mount Sinai, New York, New York; b Mark Lebwohl Center for Neuroinflammation and Sensation, Icahn School of Medicine at Mount Sinai, New York, New York; c Marc and Jennifer Lipschultz Precision Immunology Institute, Icahn School of Medicine at Mount Sinai, New York, New York; d Friedman Brain Institute, Icahn School of Medicine at Mount Sinai, New York, New York; e Allen Discovery Center for Neuroimmune Interactions, Icahn School of Medicine at Mount Sinai, New York, New York

**Keywords:** disseminated keratosis pilaris-associated itch treated with dupilumab, dupilumab, itch, keratosis pilaris, pruritus

## Introduction

Keratosis pilaris (KP) is a common, typically innocuous skin condition characterized by small, rough perifollicular papules often appearing on the extensor surfaces of the cheek, arms, buttocks, and thighs. The condition is multifactorial and is thought to stem from a genetic disorder of keratinization.^1^ KP is also frequently seen in conjunction with atopy, although it is generally a mild condition that may not require treatment. Here, we present a rare case of a male patient with atypical, generalized KP accompanied by severe pruritus that was successfully treated with dupilumab 300 mg biweekly subcutaneous (SC) injections.

## Case presentation

**Fig 1:**
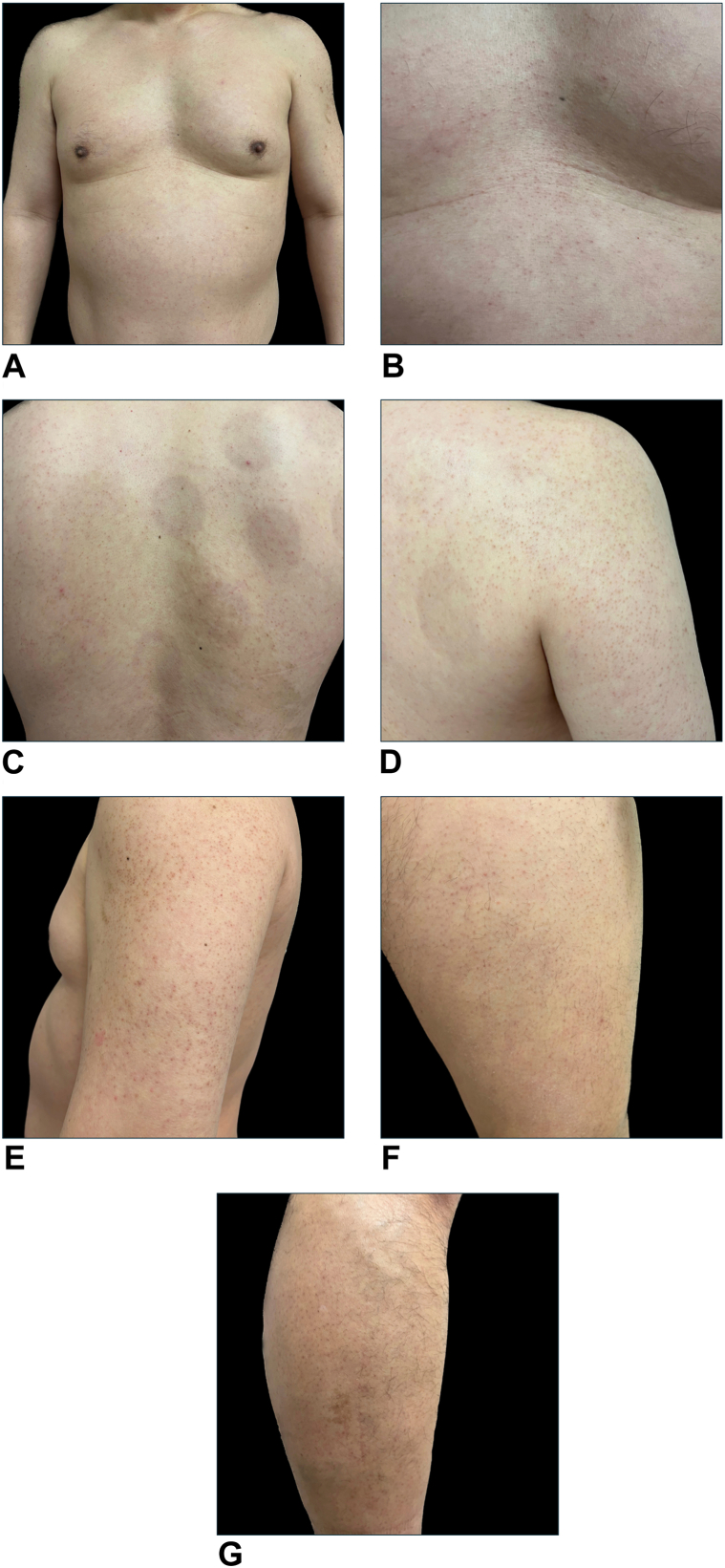
Representative clinical photographs of patient before dupilumab treatment. Diffuse papules on **(A, B)** chest, **(C)** back, **(D)** back of the arm, **(E)** upper portion of the arm, **(F)** thigh, and **(G)** lower portion of the leg.

A 43-year-old man presented with pruritic, generalized scattered small, skin-colored, keratotic, follicular papules over the torso and upper and lower extremities for 3 years (Fig 1). The lesions were initially sporadically pruritic, but he soon experienced chronic, frequent, and severe pruritus in the affected regions. He had 4 to 5 itching episodes per day. His itching worsened upon cutaneous exposure to water or moisture. Cold temperatures worsened his symptoms, whereas heat improved his itching. His 24 hour Peak Pruritus Numerical Rating Scale itch score was 10 and the itching prevented him from sleeping at night. The itching was so severe that he experienced a habit of walking around empty parking lots in the middle of the night to distract himself from the intense itching. He had no history of atopic dermatitis or other atopic conditions. His laboratory results, including routine blood cell count, basic metabolic panel, lipid panel, thyroid panel, immunoglobulin E, and liver tests, were within normal limits. Based on the patient’s skin examination, a diagnosis of KP was determined. He failed cetirizine 40 mg, hydroxyzine 25 mg, montelukast 10 mg, famotidine 20 mg twice daily, topical triamcinolone 0.1% cream, and anti-itch moisturizers. Dupilumab is a monoclonal antibody that blocks interleukin-4 receptor and, given that it is known to alleviate itch, in part, by its activity on sensory neurons,^2^ we hypothesized that dupilumab may be efficacious in disseminated KP associated with itch. Indeed, after 10 to 12 weeks of treatment with dupilumab, 600 mg SC injection followed by 300 mg SC biweekly, the patient showed notable improvement in itching. By week 14, his 24 hour Peak Pruritus Numerical Rating Scale itch score was reduced to 3 and his itching no longer prevented him from sleeping at night. Although the patient initially experienced increased alloknesis in KP-affected regions during the first 4 weeks of treatment, he began experiencing reduced aquagenic and thermal itching by week 10, and skin appearance remained consistent with the initial examination.

## Discussion

KP is typically a mild dermatologic condition of cosmetic significance only, and unlike in our patient, the condition is rarely generalized or accompanied by severe itching, although previous reports have noted generalized KP induced by medications such as nilotinib^3^^,^^4^ and testosterone injections.^5^ One report has described severe pruritus occurring in a patient with generalized unilateral KP.^6^ KP is typically diagnosed based on history and clinical features in skin examination findings. Although skin biopsies generally demonstrate follicular plugging of the hair follicle orifice, a punch biopsy is not clinically necessary for diagnosis. In many cases, KP does not require treatment, although therapies including emollients, exfoliants, topical or systemic retinoids, and laser therapy may provide symptomatic relief for cosmetic concerns.^1^

However, given our patient’s atypical and intense pruritic symptoms, a decision was made to begin treatment with dupilumab, which has received US Food and Drug Administration approval for multiple pruritic conditions such as atopic dermatitis and more recently, prurigo nodularis.^7^ Biweekly treatment with dupilumab 300 mg SC resulted in notable reduction in itch and significant improvement of this patient’s quality of life and sleep. This case serves to highlight the unmet need of patients experiencing chronic pruritus, and to our knowledge, this is the first report of a patient with itch in the setting of disseminated KP improving on treatment with dupilumab.

## Conflicts of interest

Dr Kim is founder of Alys Pharmaceuticals and KliRNA Biotech, has served as a consultant for 23andMe, ABRAX Japan, AbbVie, Almirall, Amgen, Arcutis Biotherapeutics, Arena Pharmaceuticals, argenx, AstraZeneca, Boehringer Ingelheim, Bristol-Myers Squibb, Cara Therapeutics, Clexio Biosciences, Eli Lilly and Company, Escient Pharmaceuticals, Evommune, Galderma, Genentech, GlaxoSmithKline, Granular Therapeutics, Incyte Corporation, Innovaderm Research, Janssen, Kiniksa, LEO Pharma, Maruho, Novartis, Pfizer, Recens Medical, Regeneron Pharmaceuticals, Sanofi, Septerna, Triveni Bio, Vial, and WebMD, has stock in ABRAX Japan, KliRNA Biotech, Locus Biosciences, and Recens Medical, holds a patent for the use of JAK1 inhibitors for chronic pruritus, and has a patent pending for the use of JAK inhibitors for interstitial cystitis. Author Auyeung has no conflict of interest to declare.
